# Feature Selection Using Information Gain for Improved Structural-Based Alert Correlation

**DOI:** 10.1371/journal.pone.0166017

**Published:** 2016-11-28

**Authors:** Taqwa Ahmed Alhaj, Maheyzah Md Siraj, Anazida Zainal, Huwaida Tagelsir Elshoush, Fatin Elhaj

**Affiliations:** 1 Information Assurance and Security Research Group, Faculty of Computing, Universiti Teknologi Malaysia, UTM, Johor Bahru, Johor, Malaysia; 2 Faculty of Mathematical Sciences, University of Khartoum, Khartoum, Sudan; West Virginia University, UNITED STATES

## Abstract

Grouping and clustering alerts for intrusion detection based on the similarity of features is referred to as structurally base alert correlation and can discover a list of attack steps. Previous researchers selected different features and data sources manually based on their knowledge and experience, which lead to the less accurate identification of attack steps and inconsistent performance of clustering accuracy. Furthermore, the existing alert correlation systems deal with a huge amount of data that contains null values, incomplete information, and irrelevant features causing the analysis of the alerts to be tedious, time-consuming and error-prone. Therefore, this paper focuses on selecting accurate and significant features of alerts that are appropriate to represent the attack steps, thus, enhancing the structural-based alert correlation model. A two-tier feature selection method is proposed to obtain the significant features. The first tier aims at ranking the subset of features based on high information gain entropy in decreasing order. The‏ second tier extends additional features with a better discriminative ability than the initially ranked features. Performance analysis results show the significance of the selected features in terms of the clustering accuracy using 2000 DARPA intrusion detection scenario-specific dataset.

## 1. Introduction

Intrusion detection systems (IDS) work as the "second line of defense" for computer and network systems [1]. Intrusion detection systems employ one intuitive rule that intrusive patterns are noticeable and they are unusual to regular communication. Generally, an IDS is categorized to be a host-based or network-based system depending on its monitoring capability. Host-based IDSs focus on monitoring the individual host/computer with regard to the internal activities and statuses. It cannot detect intrusion across the network [2]. Network-based IDSs (NIDSs) detect intrusions over the network by examining the packets arriving into the network. Since attempted intrusions can occur via the network, an NIDS needs to monitor different actions triggered on multiple hosts in order to build adequate evidence. In this case, NIDSs are made to handle huge data packets and communications in large networks as compared to host-based IDSs. A NIDS collects and analyses network information to check if there are actions violating security strategies [3]. The NIDS triggers alert to the network operator to take action against the suspicious activities. However, even a single NIDS generates a huge amount of alerts that overwhelms the operators [4] [5]. Most of the generated alerts have irrelevant features which result in slow training and testing correlation processes, higher resource consumption, lower accuracy and higher performance costs [6]. Furthermore, inappropriate features lead to the less accurate discovery of the attack steps. The pattern of attack steps taken by the attacker is discovered when a similar pattern of alerts are recognized and grouped. Therefore, this paper aims to identify appropriate features to achieve high accuracy in the identification of the attack steps. After that, the list of the attack steps from the alerts patterns can be determined accurately by clustering the most significant features of the alerts. The selected features are evaluated in terms of clustering accuracy. This paper proposes a 2-tier feature selection method, namely, feature ranking (first tier) and additional feature (second tier). The feature ranking tier ranks the features based on high information gain entropy while the additional feature tier provides extended additional features with better discriminative ability.

This paper is organized as follows: Section 2 provides an overview of some related research and presents the necessary background information regarding feature selection in alert correlation. Section 3 presents an overview of the proposed feature selection and discusses the experimental results. Finally, in Section 4 the paper is concluded.

## 2. Related Work

Feature selection has been widely applied in many domains, such as text categorization [7], genomic analysis [8], intrusion detection [9][10] and bioinformatics [11][12].

In a complex classification domain, such as intrusion detection, features may contain a false correlation that hinders the learning task to be processed [13]. Some features may be irrelevant and others may be redundant [14]. These extra features can increase computational time and can have an impact on the system accuracy [13]. Therefore, selecting important features from input data leads to the simplification of a problem, and faster and more accurate detection rates [9]. For this reason, alert correlation researchers have tried to select the relevant features of alerts. However, the relevant features were manually selected with different researchers selecting different features based on their knowledge and experience. For example, in alert clustering, seven different features are selected by Tjhai et al. [15] and Man et al. [16]. Tjhai et al. [15] proposed a framework that contains four phases: feature extraction, alarm aggregation, cluster analysis and classification. In the phase of feature extraction, seven alerts features (attributes) such as the number of alerts, number of signatures, port number, protocol, priority, time interval and the number of events are evaluated and chosen to represent the value of each input vector in the last phase. Man et al. [16] proposed ISODATA algorithm or the purpose of solving flood and duplicated alarms of IDS effectively. The essence of their algorithm is to generate an initial class as "seed", and then iterate clustering automatically according to some discriminate rule. DARPA 1999 is used to test their algorithm. Considering that there were a lot of useless information in the original alert, they selected a part of the attributes as their main characteristic attributes in their aggregation algorithm and represented them as a tuple containing the attributes: alert id, alert type, SrcIP, DestIP, SrcPort, DestPort, and Time. Mohamed et al. [17] extracted three attributes from the alerts (destination IP, signature type or id and timestamp) and applied these attributes to the MD5 hash function. The MD5 generates a unique hash value that is used for the initial clustering process. Meanwhile, Siraj [18] proposed a novel hybrid clustering model based on Improved Unit Range (IUR), Principal Component Analysis (PCA) and unsupervised learning algorithm (Expectation Maximization) to aggregate similar alerts and to reduce the number of alerts. Three different attributes (source port, destination port, and alert type) from the DARPA 2000 dataset were selected and represented as a vector. Shittu et.al. [19] propsed A comperhensive System for Analysing Intrusion Alerts (ACSAnIA). it contains seven components which are: (1) Offline Correlation (2) Online Correlation (3)Meta alert Comparison (4) Meta–alert Prioritisation (5)Meta–alert Clustering (6) Attack Pattern Discovery and (7) Reporting System. The ACSAnIA system uses six of the alerts attributes where as: alert’s timestamp, source IP, source port, destination IP, destination port and intrusion type. Furthermore, based on Ramaki et al.[20] an efficient framework for alert correlation in Early Warning Systems (EWSs) is proposed. An important process in EWSs is the analysis and correlation of alerts aggregated from the installed sensors. The authors mentioned that an alert consists of some features based on specific attributes of network traffic. The most important features that are used for the their alert correlation process are: source IP address, destination IP address, source and destination port numbers, intrusion type or alert type, attack severity and timestamp.

The literature review indicates that there are no standards or specific features used in alert clustering; thus, every researcher selects a different number of feature subsets based on their own experience. Therefore, this paper focuses on applied automated feature selection that improves clustering accuracy and presents accurate attack steps.

## 3. Feature Selection

**Fig 1:**
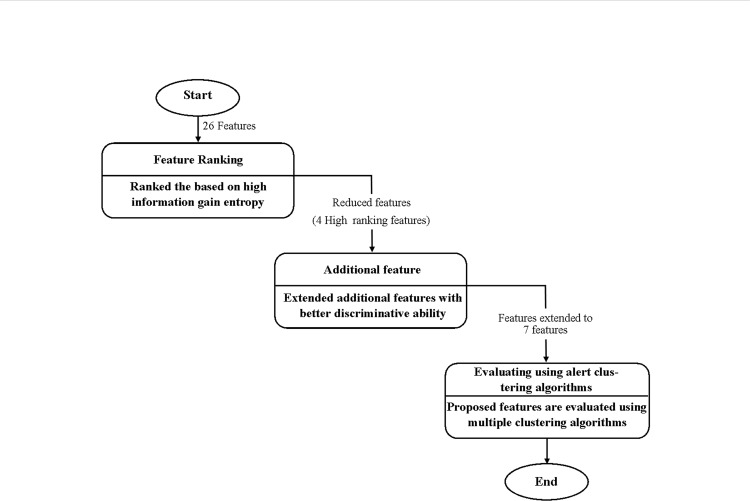
Two-Tier Feature Selection Procedure.

The reason for selecting the important and significant features is to represent the attack steps from the alerts pattern correctly and improve the accuracy of the Structural based Alert Correlation (SAC). This section describes the two-tier feature selection, i.e., feature ranking and additional feature. The feature ranking stage employs Information Gain algorithm (IG) that uses a filtering approach. The stage aims at ranking subsets of features based on high information gain entropy in decreasing order. Meanwhile, the additional feature stage is based on the work of Ren et. al. [21] where they mention that identifying relationships between alerts essentially needs to analyses the alerts' attributes, and extracting the basic attributes may not be sufficient to fully discover the relationship between the alerts. Therefore, the aim of this stage is to extend additional features that contribute to the relationship between alerts with a better discriminative ability than the initially ranked features. Fig 1 shows the feature selection procedure that is adopted in this research. The effectiveness of the reduced feature subsets was evaluated on SAC.

### 3.1 Feature Ranking

Ranking methods are used due to their simplicity and the fact that good success has been reported for practical applications [22]. A suitable ranking criterion is used to score the variables and a threshold is used to remove variables below the threshold. The basic property of feature ranking is to identify the relevance of the features. It essentially states that if a feature is to be relevant it can be independent of the input data but cannot be independent of the class labels, i.e., the feature that has no influence on the class labels can be discarded [22]. The main reason behind the application of feature ranking in this study is based on this property, which ranks the feature that has an influence on the class labels.

Hyper alerts, as well as low-level alerts, can be distinguished based on the type of alert that denotes a certain attack class/step [21]. Furthermore, the absence of truth labeled in DRAPA 2000 datasets directed this research towards proposing alert types as class labels to represent accurate attack steps. Therefore, based on the feature ranking property, high ranking features are most relevant and significant for alert types.

**Fig 2:**
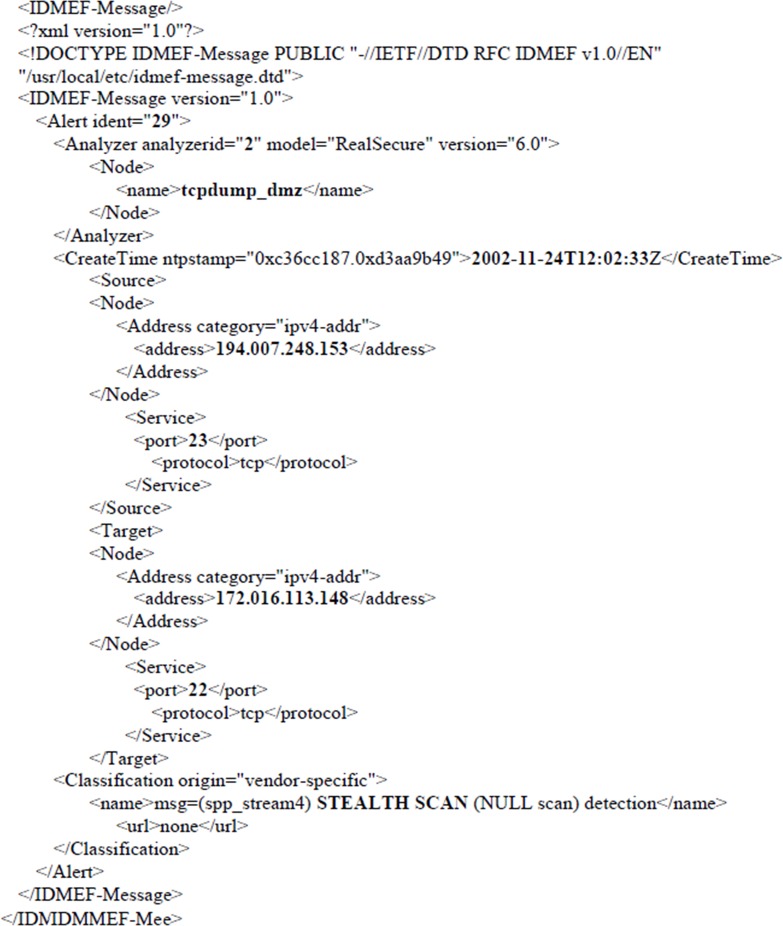
IDMEF alert format in an XML document.

As mentioned earlier, the feature ranking was implemented using Information Gain (IG). IG is frequently employed as a term-goodness criterion in the field of machine learning [23]. It is measured based on the entropy of a system, i.e., of the degree of disorder of the system. Therefore, the entropy of a subset is a fundamental calculation to compute IG. For feature ranking purpose, IG is implemented in all four files of datasets. Twenty-six features are applied to IG for feature ranking. This study manually identified some of those features that have network meaning and is based on the XML file presented in Fig 2.

Referred to Fig 2 the alert is uniquely identified by the alert ident feature. The source and target feature describe the node and service of the sender and the receiver respectively. The node contains the IP address and its category, while service holds the port number and its corresponding protocol. The alert type is given by the Classification name feature. Specifically, this alert simply represents a stealth scan attack via port 23 from 194.007.248.153 to 172.016.113.148 via port 22. Based on this information, eight features have been identified from the XML documents as listed in Table 1 and the rest have been labelled without network meaning. Table 2 is an example of all features in DMZ network for scenario one.

**Table 1:** Attributes of an alert extracted from the XML document.

Extracted Feature	New Labelled Feature	Description
Alert ident	*AlertID*	The number of alerts in a network session
Analyzerid	*SensorID*	Name or identification index of NIDS
CreateTime	DetectTime	Time of the alert occurred
Source/Node/Address	*SourceIPAddress*	IP address of a sender
Target/Node/Address	*DestinationIPAddress*	IP address of a receiver
Source/Service/Port	*SourcePort*	Sender’s Port number
Target/Service/Port	*DestinationPort (DestPort)*	Receiver’s Port number
Classification/name	*AlertType*	The alert type based on signature files

**Table 2:** All features of DRAPA 2000 datasets.

Label	Network Data Features	Label	Network Data Features
A_ID	AlertID	Des_Mc_address	Destination MAC Address
X1	not identified in xml file	S_Mc_address	SourceMAC Address
X2	not identified in xml file	X7	not identified in xml file
X3	not identified in xml file	X8	not identified in xml file
X4	not identified in xml file	X9	not identified in xml file
Date	Detect Date	X10	not identified in xml file
Time	DetectTime	Priority	Priority
S_port	SourcePort	Sensor ID	Sensor ID
Source _IP	SourceIPAddress	A_ Type	Alert type
D_port	DestinationPort	X11	not identified in xml file
Target_IP	Target IPAddress	X12	not identified in xml file
X5	not identified in xml file	X13	not identified in xml file
X6	not identified in xml file	X14	not identified in xml file

**Fig 3:**
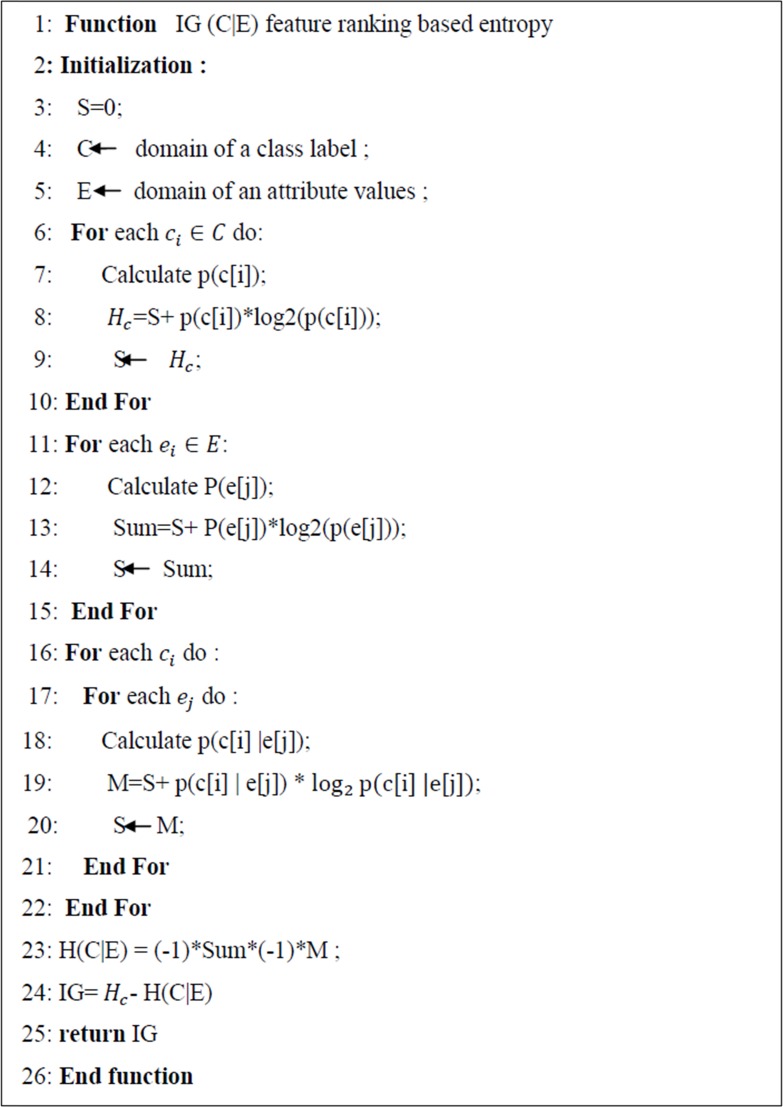
Information Gain algorithm.

IG looks at each feature in isolation, computes its information gain and measures how important and relevant it is to the class label (alert type). Computing the information gain for a feature involves computing the entropy of the class label (alert type) for the entire dataset and subtracting the conditional entropies for each possible value of that feature. The entropy calculation requires a frequency count of the class label by feature value. In more details, all instances (alerts) are selected with some feature value v, then the number of occurrences of each class within those instances are counted, and the entropy for v is computed. This step is repeated for each possible value v of the feature. The entropy of a subset can be computed more easily by constructing a count matrix, which tallies the class membership of the training examples by feature value. The algorithm of IG implementation is given in Fig 3.

#### 3.1.1 Results on Feature Ranking

Tables 3, 4, 5 and 6 show that for each dataset file, the different set of features are ranked differently in decreasing order based on their relevance to class label. The reason behind this variation is that the DDoS attack has five attack phases, in each phase different attack steps occurred. The rank score measures how much each feature relates and contributes to class label. These tables indicate that A_ID, D_port, Priority and S_port which are: alert id, destination port, priority and source port (as highlighted in bold) have obtained the same sequence of order with higher scores than other features in all files of datasets. Therefore, we can conclude that those four features are more related and influential to alert type (class label).

**Table 3:** Feature ranking using IG on DMZ 1 DARPA 2000 dataset.

**Features**	**Scores**	**Features**	**Scores**
**A_ID**	2.418	S_Mc_address	0.34
**D_port**	1.746	Des_Mc_address	0.159
**Priority**	1.062	X2	0.095
**S_port**	0.896	Date	0.094
X4	0.852	X8	0
X5	0.849	X9	0
Time	0.772	X12	0
X3	0.749	X7	0
Source _IP	0.633	X11	0
X6	0.559	X10	0
Target_IP	0.465	X13	0
Sensor ID	0.426	X14	0
X1	0.426		

**Table 4:** Feature ranking using IG on Inside 1 DARPA 2000 dataset.

**Features**	**Scores**	**Features**	**Scores**
**A_ID**	2.367	Sensor ID	0.267
**D_port**	1.815	X1	0.267
**Priority**	0.977	X14	0.209
**S_port**	0.575	X2	0
X5	0.555	X11	0
Time	0.543	X12	0
X4	0.542	X13	0
X3	0.532	Date	0
Target_IP	0.469	X7	0
Source _IP	0.455	X8	0
X6	0.453	X9	0
S_Mc_address	0.389	X10	0
Des_Mc_address	0.276		

**Table 5:** Feature ranking using IG on DMZ 2 DARPA 2000 dataset.

**Features**	**Scores**	**Features**	**Scores**
**A_ID**	2.2	Sensor ID	0.077
**D_port**	1.561	X1	0.077
**Priority**	1.049	X11	0.
**S_port**	0.674	X2	0
Target_IP	0.663	X12	0
Source _IP	0.538	X13	0
X6	0.528	X10	0
X3	0.303	Date	0
Time	0.302	X7	0
X5	0.302	X8	0
X4	0.291	X9	0
S_Mc_address	0.163	X14	0
Des_Mc_address	0.16		

**Table 6:** Feature ranking using IG on Inside 2 DARPA 2000 dataset.

**Features**	**Scores**	**Features**	**Scores**
**A_ID**	2.373	X1	0.166
**D_port**	1.741	Sensor ID	0.166
**Priority**	1.08	X8	0
**S_port**	0.52	X2	0
X6	0.518	Date	0
S_Mc_address	0.418	X11	0
Target_IP	0.344	X12	0
Des_Mc_address	0.334	X7	0
Source _IP	0.206	X9	0
X3	0.181	X10	0
Time	0.178	X13	0
X4	0.178	X14	0
X5	0.178		

#### 3.1.2 Evaluation Performance on Feature Ranking

To evaluate the significance of these ranked features on clustering accuracy that present the attack steps and to find the best algorithm that produces the highest clustering accuracy, three clustering algorithms have been applied both before and after the feature selection method. The algorithms are K-Means, EM, and Hierarchical. Clustering refers to unsupervised learning and for that reason, it has no priori data set information. Therefore, many different cluster validity methods have been proposed without prior class information, these are named internal validity. In contrast, there is a number of external cluster validity for which a priori knowledge of dataset information is required. As noted before, the absence of truth labels in DARPA 2000 datasets for evaluating structured (cluster) based alert correlation leads this research to propose a single class label that is an alert type for external validation of the clustered alerts.

**Fig 4:**
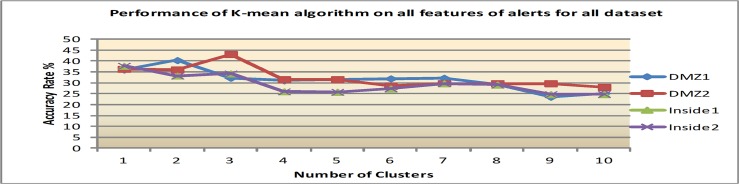
Results of K-means with varying number of clusters.

**Fig 5:**
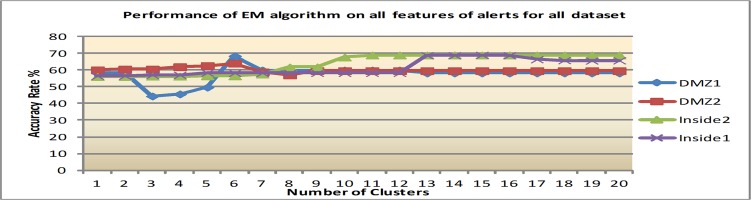
Results of EM with varying number of clusters.

**Fig 6:**
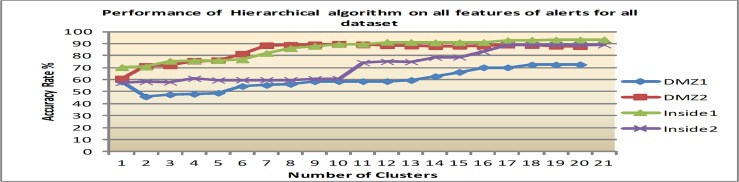
Results of Hierarchical with varying number of clusters.

Firstly, the clustering algorithms were performed on the original features, a total of 26, for all datasets before applying IG for feature ranking. The average accuracy rates (AR), which include the percentage of alerts that are accurately clustered, are reported in Figs 4, 5 and 6.

1) Performance using K-means algorithm

Fig 4 presents that K-means algorithm achieves the best result (37% to 43%) with two and three clusters. In average, for all dataset, the best performance of K-means algorithm is 39.7% AR at 2 clusters. After that, the accuracy is slowly downgraded when the number of clusters is increased. The graph in all figures shows that the performance of K-means is poor, it gives in average AR of 39.7%. In addition, the drawback of this algorithm is that there is no efficient and universal method for identifying the initial partitions; and the centroids are varied with different initial points that lead to different results in different iterations.

2) Performance using EM algorithm

Within the EM algorithm, the highest accuracy (64% to 68.9%) is obtained when the number of clusters is between 7 and 16. When the number of clusters is more than this range, the performance of EM decreases. In average for all dataset, the EM's best performance is 67.5%. The result of EM algorithm is shown in Fig 5. The results show that EM is about 27.8% better than K-means. However, when the number of clusters is increased there is a possibility of EM to produce clustering and an incorrect group of alerts pattern. Hence, the experiments were continued with another algorithm.

3) Performance using Hierarchical algorithm

Meanwhile, Fig 6 shows that Hierarchical cluster gives a slight improvement in its curve compared to EM. It produces a consistent result in the range of (72.3% to 93.2%) when the number of clusters equals the number of alerts type (class label) which are 17 clusters and above in all datasets. In average for all dataset, the hierarchical has the best performance 85.6% AR. With this, hierarchical performs better than K-means and EM with approximately 45.9% and 18.1% improvements respectively. The justification for adopting Agglomerative hierarchical cluster is that the algorithm starts with each data point (feature) as a separate class and then each step of the algorithm involves merging two clusters that are most similar. This point is very useful in alert clustering to identify the accurate attack step.

Table 7 details the best clustering Accuracy Rate (AR) produced by, K-means, and EM and Hierarchical algorithms on all datasets before feature selection. The details are based on results from Figs 4–6.

**Table 7:** Summary on AR using K-means, EM and Hierarchical algorithm on all datasets before feature selection.

Datasets	k	Accuracy of K-means	k	Accuracy ofEM	k	Accuracy of Hierarchical
DMZ1	3	43.7	7	68.3	19	72.3
Inside1	2	37.7	7	64	17	88.2
DMZ2	4	43.1	16	68.9	22	93.2
Inside2	2	37.7	12	68.9	20	88.9
Mean	3	40.5	11	67.5	20	85.6

**Fig 7:**
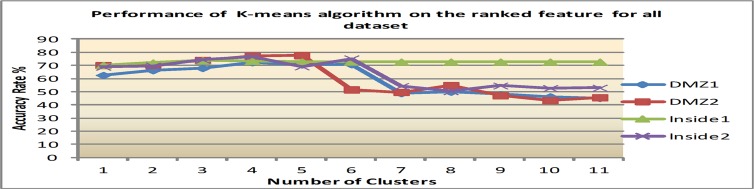
Results of K-means after feature ranking.

**Fig 8:**
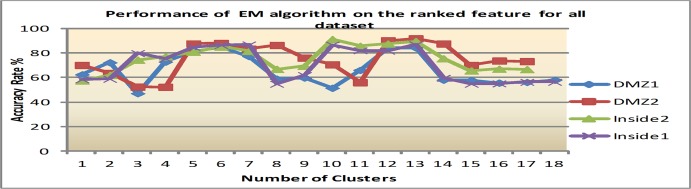
Results of EM algorithm after feature e ranking.

**Fig 9:**
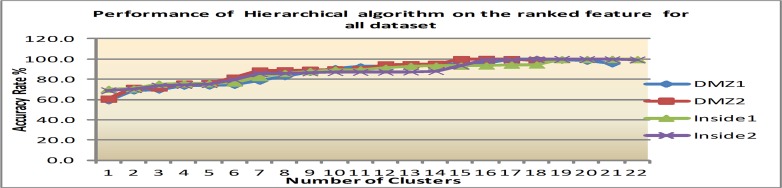
Results of Hierarchical after feature ranking.

Secondly, with the feature ranking that is mentioned above, four significant features which are: alert id, destination port, priority and source port are applied to the same clustering algorithms. The reason for this is to empirically prove that the ranked features improve clustering performance. Figs 7–9 show clustering accuracy rate with varying number of clusters to find optimum results.

1) K-means performance after feature ranking

In regards to K-Means, this study found that a range of 4 to 6 clusters yield the best performance, namely that of 75.1 percent AR for all datasets. After that, any increase in the cluster numbers implies a decrease in the clustering accuracy. There is a 35.4% improvement when compared to cluster performance before feature ranking. The result of K-means after feature selection is shown in Fig 7.

2) EM performance after feature ranking

88.5% has been obtained in average for all dataset within the EM algorithm when the number of clusters is between 11 and 14. The results show that after feature ranking there is an improvement of about 22.2%. The detailed result of EM after feature ranking is presented in Fig 8.

3) Hierarchical performance after feature ranking

In this experiment, the hierarchical clustering algorithm obtained the highest accuracy rate when the number of clusters equaled the number of alerts type in all datasets. As shown in Fig 9, with DMZ1, 100% AR is obtained when the number of clusters equals the number of alert type (19). Therefore, 19 clusters of attack steps are presented. If the number of clusters is more than 19, the clustering accuracy slowly downgrades. Furthermore, the clustering algorithm gives a high accuracy of 100% with 17, 20 and 22 number of clusters in DZM2 for inside1 and inside2 datasets respectively. Consequently, the improvement of hierarchical clustering algorithm after feature ranking is about 14.6%.

Table 8 summarizes the best clustering accuracy offered by K-means, EM and Hierarchical algorithms on all datasets after feature ranking. The details are based on results from Figs 7–9. The hierarchical algorithm gives the best result among the studied algorithms.

**Table 8:** Summary of clustering accuracy using K-means, EM and Hierarchical algorithm on all datasets after feature ranking.

Datasets	k	Accuracy of K-means	k	Accuracy of EM	k	Accuracy of Hierarchical
DMZ1	5	72.2	13	84.3	19	100
Inside1	4	73.7	14	86.8	17	100
DMZ2	6	77.6	14	91.7	22	100
Inside2	5	76.8	11	91.2	20	100
Mean	5	75	13	88.5	20	100

The reported empirical results on the investigated clustering algorithms lead to several observations and discussions:

1) Observation 1: Hierarchical cluster is a suitable candidate to cluster alerts in the structural-based AC because the algorithm starts with each feature as a separate class or cluster and then merges the clusters or the classes that are more similar. This mean that, each ranked feature (i.e., alert id, destination port, priority and source port) has its own cluster. After that, each cluster of a feature is compared to a class label (alert type) that denotes a certain attack class/step to measure the similarity and relevance between the cluster feature and the class label. Therefore, when the cluster of alert id feature compares to a class label (alert type), the algorithm obtains 100% AR because alert id is a dominant feature to alert type. However, this algorithm considers alert id as the main feature and the validation of the algorithm is based on this feature regardless of other features. For this reason, the investigation of other clustering algorithms to evaluate the significance of the other three features is needed.

2) Observation 2: The empirical results prove that the ranked features which are: alert id, destination port, priority and source port yields the best performances for EM and K-means algorithms, namely that of 88.5% and 75.1% AR respectively for all datasets. However, this improvement is still moderate, and according to Ren et al. [21], selecting the basic alert features may not be sufficient to fully discover these patterns. Furthermore, this research seeks to find accurate features that offer high cluster accuracy and represent the step of the attack accurately. Thus, additional features from the available dataset features which are useful in alert correlation are derived.

The above discussions motivate further investigations and experiments to improve the clustering accuracy. The experiments involve the use of three additional features along with ranked features, to produce an enhanced SAC model. The corresponding results are reported in the next section.

### 3.2 Additional Feature

IDS alerts features capture intrinsic attack characteristics that are mainly for identifying attack strategy such as the IP address of an alert, its port number, and time when the alert is triggered [21]. While the values of these features are the same for low-level alerts grouped on hyper-alert (except time), their values differ among the hyper alerts of the same type. At the same time, feature values of hyper alerts share common patterns that allow describing the hyper-alert type [21]. Therefore, selecting the basic alert features may not be sufficient to fully discover these patterns. For this reason, additional features from the available dataset features which are useful in alert correlation were derived.

Source_IP, target_IP and time have been added as additional features because the source and destination IP addresses are the key of correlation [24]. Also, the time attributes can help to associate and cluster alerts that occur in short intervals [25]. It is concluded that alert ID, source port, destination port, source IP, destination IP, priority and time are the most significant features that are needed in alert correlation as described in Table 9.

**Table 9:** The description of significant features of DARPA 2000 dataset.

**Label**	**Corresponding Features**	**Description of Features**
**A_ID**	Alert ID	Unique identifier of alert
**D_port**	Destination Port	Receiver’s Port number
**Priority**	Priority	Describes the Severity of alerts
**S_port**	Source Port	Sender’s port number
**Source _IP**	Source IP address	IP address of sender
**Target_IP**	Target IP address	IP address of a receiver
**Time**	Time	The time when alert is generated

**Fig 10:**
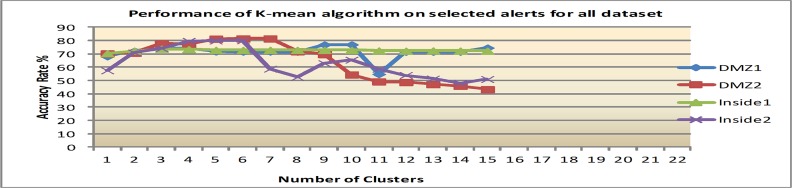
Results of K-means based on the seven selected features.

**Fig 11:**
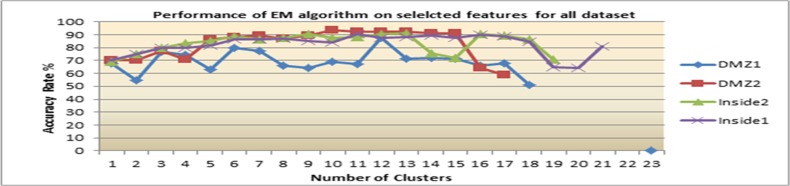
Results of EM based on the seven selected features.

**Fig 12:**
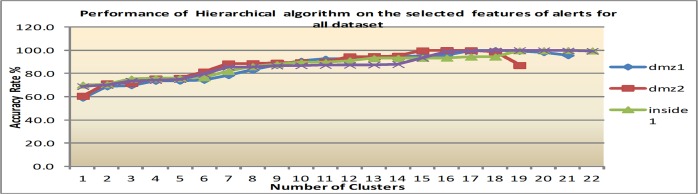
Results of Hierarchical based on seven selected features.

Fig 10, Fig 11 and Fig 12 show the performance of the clustering accuracy of K-means, EM and Hierarchical algorithms based on the seven selected features. They show that for each clustering algorithm tested K-means, EM, and Hierarchical, the selected features contribute superior results with an average of 77.9%, 90.6% and 100% AR respectively. As mentioned before, the 100% AR obtained by the hierarchical algorithm is due to the dominant feature of alert id. K-means and EM empirical results prove that the additional features improve the clustering accuracy in the average of 2.8% and 2.1% respectively. The Summary of AR using K-means, FCM and EM algorithm on all datasets is presented in Table 10.

**Table 10:** Summary on AR using K-means, FCM and EM algorithm on all datasets.

	K-means					EM					Hierarchical				
	DMZ1	Inside1	DMZ2	Inside2	Mean	DMZ1	Inside1	DMZ2	Inside2	Mean	DMZ 1	Inside1	DMZ2	Inside2	Mean
Raw Data	43.7	37.7	43.1	37.7	40.5	68.39	64	68.9	68.9	67.5	72.3	88.2	93.2	88.9	85.6
Rank Feature	72.2	73.7	77.6	76.8	75	84.3	86.8	91.7	91.2	88.5	100	100	100	100	100
Selected Feature	76.8	73.7	81.4	79.9	77.9	87.5	90.4	93.4	91.2	90.6	100	100	100	100	100

## 4.Discussion

In this paper, the practical results have confirmed that using the proposed features (ranking + additional features) gives significantly better clustering performance for representing attack step. As mentioned before each alert cluster represents an attack step of multi-stages attacks. Table 11 represents the attack steps which are listed alphabetically and a number of alerts for each cluster is shown in the brackets. Based on the hierarchical clustering algorithm, the number of attack steps discovered in each dataset is 19, 22, 17 and 20 respectively. A brief meaning of each attack step is described in Table 12. We observe that the Email_Ehlo is the highest number of alerts in all dataset. This indicates that the attacker is trying hard to access information as much as he/she can from the Simple Mail Transfer Protocol (SMTP) configurations. Since SMTP is used to transfer e-mail messages between computers, this attempt is most likely to find a vulnerable path that the attacker can use to send harmful messages or files. When such intrusion is successful, a lot of Email_Almail_Overflow are detected, indicating that the attacker is trying to overflow the email buffer. Other clusters which have a large number of alerts are TelnetTerminaltype alerts, which indicate the beginning of a telnet session using the reported Terminal type has been detected. Furthermore, a high volume of alerts also precedes the File Transfer Protocol (FTP), for example, FTP_Pass, FTP_Syst, FTP_Put, and FTP_User. Based on Table 12, they are related to a standard network protocol which used to copy a file from one host to another over a TCP/IP-based network, such as the Internet. Additionally, FTP is built on a client-server architecture and utilizes separate control and data connections between the client and the server. Although users need to authenticate themselves using a clear-text sign-in protocol, sometimes they can connect anonymously if the server is configured to allow it. This is most probably the best reason for the attacker to exploit the FTP at the destination host.

**Table 11:** List of attack steps (clusters) discovered on all dataset.

Cluster index	DMZ1 (19 clusters, 886 alerts)	Inside1 (22 clusters, 922 alerts)	DMZ2 (17) clusters, 425 alerts)	Inside2 (20 clusters, 489 alerts)
**1**	Admind (38)	Admind (17)	Admind (2)	Admind (4)
**2**	Email_Almail_Overflow (40)	Email_Almail_Overflow (38)	Email_Almail_Overflow (23)	Email_Almail_Overflow (22)
**3**	Email_Debug (2)	Email_Debug (2)	Email_Ehlo (253)	Email_Ehlo (272)
**4**	Email_Ehlo (515)	Email_Ehlo (522)	Email_Turn (1)	Email_Turn (1)
**5**	FTP_Pass (36)	FTP_Pass (49)	FTP_Pass (20)	FTP_Pass (27)
**6**	FTP_Syst (34)	FTP_Syst (44)	FTP_Put (1)	FTP_Put (2)
**7**	FTP_User (36)	FTP_User (49)	FTP_Syst (16)	FTP_Syst (18)
**8**	HTTP_Cisco_Catalyst_Exec (2)	HTTP_Cisco_Catalyst_Exec (2)	FTP_User (20)	FTP_User (27)
**9**	HTTP_Java (8)	HTTP_Shells (15)	HTTP_ActiveX (1)	HTTP_ActiveX (1)
**10**	HTTP_Shells (15)	HTTP_Java (8)	HTTP_Cisco_Catalyst_Exec (5)	HTTP_Cisco_Catalyst_Exec (5)
**11**	Rsh (16)	Mstream_Zombie (6)	HTTP_Java (30)	HTTP_Java (30)
**12**	Sadmind_Amslverify_Overflow (32)	Port_Scan (1)	Sadmind_Amslverify_Overflow (2)	Mstream_Zombie (3)
**13**	Sadmind_Ping (6)	RIPAdd (1)	SSH_Detected (2)	Port_Scan (1)
**14**	SSH_Detected (8)	RIPExpire (1)	TCP_Urgent_Data (2)	RIPAdd (1)
**15**	TCP_Urgent_Data (8)	Rsh (17)	TelnetEnvAll (1)	Sadmind_Amslverify_Overflow (4)
**16**	TelnetEnvAll (1)	Sadmind_Amslverify_Overflow (14)	TelnetTerminaltype (45)	Stream_DoS (1)
**17**	TelnetTerminaltype (87)	Sadmind_Ping (3)	TelnetXdisplay (1)	TCP_Urgent_Data (1)
**18**	TelnetXdisplay (1)	SSH_Detected (4)		TelnetEnvAll (2)
**19**	UDP_Port_Scan (1)	Stream_DoS (1)		TelnetTerminaltype (65)
**20**		TelnetEnvAll (1)		TelnetXdisplay (2)
**21**		TelnetTerminaltype (126)		
**22**		TelnetXdisplay (1)		

**Table 12:** Description of attack steps based on RealSecure Signatures Reference Guide Version 6.0 (Internet Security Systems.

Alert Cluster	Description
Admind	If it is used with insecure authentication, an attacker could compromise the computer and add user accounts.
Email_Almail_Overflow	It can overflow a buffer (e.g. email) and the attacker can execute arbitrary code.
Email_Debug	An attempt to initiate a root-level shell on the target host.
Email_Ehlo	An attempt to determine the configuration information on SMTP daemons.
Email_Turn	An attempt to pick up mail intended for other hosts. Since only very old versions of Sendmail are vulnerable to this attack, it is a false positive
FTP_Pass	The FileTransfer Protocol (FTP) passes a plaintext password across the network to allow a user has access to the files.
FTP_Put	The FTP uses a PUT (technically STOR) command in order to transfer the files.
FTP_Syst	An attempt to know the type of server’s operating system to exploit other vulnerabilities likely to be present.
FTP_User	It records the username on the FTP server of the person transferring files.
HTTP_ActiveX	ActiveX is a Web technology that can be used to execute a local command (e.g. to shut down) the computer.
HTTP_Cisco_Catalyst_Exec	An attempt to view the configuration file and obtain user passwords.
HTTP_Java	In a Java enabled Web browser, the browser may access files that contain Java code from remote Web sites.
HTTP_Shells	It is considered a bad security practice to put shell interpreters (e.g. sh) in the *cgi-bin* directory. This vulnerability is false positive.
Mstream_Zombie	The mstream program is a distributed denial of service tool based on the *stream*.*c* attack.
Port_Scan	A portscan is an attempt by an attacker to determine what services are running on a system by probing each port for a response.
RIPAdd	An attempt to gain access by loading false information into the network routing tables.
RIPExpire	When a RIP entry is being timed out, one of the networks is about to be marked as unreachable.
Rsh	Rsh uses very weak authentication mechanisms, and has historically been frequently used by attackers to penetrate systems.
Sadmind_Amslverify_Overflow	An attempt to overflow a buffer in the amsl_verify() function and execute arbitrary code with root privileges.
Sadmind_Ping	An attempt to scan a network for potentially vulnerable systems.
SSH_Detected	The Secure Shell (SSH) protocol is an encrypted alternative to other interactive login protocols like rsh, rlogin, and telnet.
Stream_DoS	The *stream*.*c* attack is a denial of service attack designed to crash a vulnerable system by sending a flood of spoofed TCP packetswith the ACK flag set to random destination ports on the host.
TCP_Urgent_Data	An attacker could misuse Out of band (OOB) data to evade IDS or execute some Windows denial of service attacks.
TelnetEnvAll	An attempt to allow users to pass environment variables from the remote system.
TelnetTerminaltype	The beginning of a telnet session using the reported *Terminaltype* has been detected.
TelnetXdisplay	An XDisplay that is different than the source IP address may indicate an attack.
UDP_Port_Scan	An attempt to scan UDP ports to reveal listening client or server processes before performing an attack.

**Fig 13:**
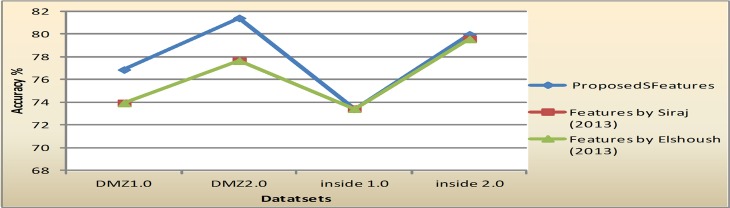
Comparison on accuracy performance of K-means in all datasets.

**Fig 14:**
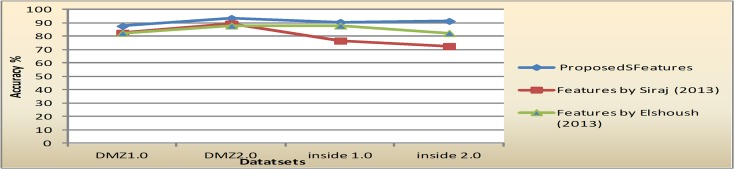
Comparison on accuracy performance of EM in all datasets.

Furthermore, For comparison with the proposed features, the features subsets selected by Siraj [18] and features subsets selected by Elshoush [26] were compared. The metrics for comparison is the clustering accuracy. Siraj [18] claimed that three significant features were enough to cluster alerts; these are Alert type, Source port, and destination port. While Elshoush [26] suggested seven features to cluster alerts namely: EventID, times, SrcIPAddress, DestPort, DestIPAddress, OrigEventName, and SrcPort. Table 13 shows the performance comparison among these feature subsets. The second row of Table 13 is the clustering accuracy of K-means and EM of all datasets based on the features subset proposed by Siraj[18]. Meanwhile, the final row is the accuracy rate of the same clustering algorithms for the features that were reported by Elshoush [26]. Performance comparison in Table 13 shows that, overall, the selected features proposed in this research give better clustering results compared to the features proposed by [18] and [26]. Fig 13 and Fig 14 illustrate the comparison in graphical formats.

**Table 13:** Performance comparison with other feature subsets.

	K-means					EM				
	DMZ1	Inside1	DMZ2	Inside2	Mean	DMZ1	Inside1	DMZ2	Inside2	Mean
**Proposed Features**	76.8	73.7	81.4	79.9	**77.9**	87.5	90.4	93.4	91.2	**90.6**
**Proposed Features by Siraj (2013)**	73.9	73.4	77.6	79.5	**76.1**	82.3	76.5	89.1	72.3	**80.1**
**Proposed Features by** Elshoush **(2013)**	73.9	73.4	77.6	79.5	**76.1**	82.3	87.7	87.7	82.2	**84.9**

In conclusion of the performance validation and benchmark of the proposed features, some observations have been outlined:

1. The Alert ID, Destination Port, and Source Port, which are high impact features in ranking, are the same features used in [18], however, giving lower accuracy than the proposed selected features.
2. Source IP address, Destination IP address and time which are proposed as additional features with the proposed features ranking are the same features used in [26]. Also, [26] gives better performance than [18] which used the same ranking features only.

## 5. Conclusion

Clustering and finding relationships between alerts is an important issue since alerts are not significant if they are isolated. The pattern of attack steps taken by the attacker is discovered when the similar pattern of alerts are recognized and grouped based on proper features. Different features were selected manually by previous researchers based on their knowledge experience which lead to less accurate in the identification of attack steps and inconsistent performance of clustering accuracy. This paper focuses on presenting accurate attack steps by proposing a 2-tier feature selection method to select appropriate and significant features. The selected features are evaluated in terms of clustering accuracy. The empirical results show that the selected features can significantly identify accurate attack steps and improve the overall clustering performance.
